# Development of Bio-Based Films and 3D Objects from Apple Pomace

**DOI:** 10.3390/polym11020289

**Published:** 2019-02-08

**Authors:** Jesper Gustafsson, Mikael Landberg, Veronika Bátori, Dan Åkesson, Mohammad J. Taherzadeh, Akram Zamani

**Affiliations:** Swedish Centre for Resource Recovery, University of Borås, 50190 Borås, Sweden; GustafssonJesper@outlook.com (J.G.); mikaellandberg744@hotmail.com (M.L.); dan.akesson@hb.se (D.Å.); mohammad.taherzadeh@hb.se (M.J.T.); akram.zamani@hb.se (A.Z.)

**Keywords:** apple pomace, bio-based film, biomaterials, compression molding, fiberboard, solution casting

## Abstract

Extensive quantities of apple pomace are generated annually but its disposal is still challenging. This study addresses this issue by introducing a new, environmentally-friendly approach for the production of sustainable biomaterials from apple pomace, containing 55.47% free sugars and a water insoluble fraction, containing 29.42 ± 0.44% hemicelluloses, 38.99 ± 0.42% cellulose, and 22.94 ± 0.12% lignin. Solution casting and compression molding were applied to form bio-based films and 3D objects (i.e., fiberboards), respectively. Using glycerol as plasticizer resulted in highly compact films with high tensile strength and low elongation (16.49 ± 2.54 MPa and 10.78 ± 3.19%, respectively). In contrast, naturally occurring sugars in the apple pomace showed stronger plasticizing effect in the films and resulted in a fluffier and connected structure with significantly higher elongation (37.39 ± 10.38% and 55.41 ± 5.38%, respectively). Benefiting from the self-binding capacity of polysaccharides, fiberboards were prepared by compression molding at 100 °C using glycerol or naturally occurring sugars, such as plasticizer. The obtained fiberboards exhibited tensile strength of 3.02–5.79 MPa and elongation of 0.93%–1.56%. Possible applications for apple pomace biomaterials are edible/disposable tableware or food packaging.

## 1. Introduction

The widespread use of synthetic plastics is leading to significant, well-documented impacts on the environment, and replacing them with bio-based alternatives may mitigate the effects of pollution as well as greenhouse gas emissions. This has led to the development of a rich and diverse field of research in bioplastic production. Biopolymers from agricultural resources, such as starch [1], cellulose [2], proteins [3], and pectin [4], are among the predominant materials used for bioplastic production.

The latest research [5,6,7,8,9,10] focuses on by-products and waste materials of the fruit and vegetable industries for bioplastic production. An interesting raw material for bioplastics production is apple pomace. Worldwide, approximately 70 million tons of apples are produced annually [11]. Apple pomace represents 25%–30% of the original apple weight [12,13]. Therefore, millions of tons of apple pomace are generated every year in the world as a byproduct of juice, cider, or wine production. The acidic characteristics of apples, with their high sugar and low protein content, makes pomace unsuitable for landfilling and animal feedstock [11,12,13]. This residue has a high moisture and biodegradable organic content which can be used for bioplastic production. Apple pomace consists mainly of cellulose (7%–44%), starch (14%–17%), pectin (4%–14%), and insoluble lignin (15%–20%) [14].

Films and 3D objects are among the category of biomaterials with high demands in the market. Solution casting, in which biopolymer solutions are applied on a surface and dried, is a very effective method for production of thin films. One possible way to make thin films from lignocellulosic materials is to dissolve the cellulose or lignin part before casting, and several solvents have been suggested [15,16,17,18]. Recently, we have developed pectin-based thin films from citrus waste using solution casting method [5]. The method was based on pectin dissolution along with dispersion of cellulose fibers in citric acid solution, which was then dried to thin films. Compared to other studies [6,8,10,19], in which fruit or vegetable wastes were used for bio-based film fabrication with the solution casting method, using hydrochloric acid solution [8], trifluoroacetic acid solution [6,19], or heptane [10], the pectin-cellulose bio-based film from orange waste have applied so far, the less harmful solvent, such as citric acid solution. This method has not been applied on apple pomace before.

Molding usually has lower energy demand compared to casting. For non-thermoplastics biopolymers, such as proteins, heat compression molding has been applied to produce bioplastic items [20]. Lignocellulosic materials have shown interesting self-binding capacity under pressure and heat, which opened up opportunities for preparation of binder-less bio-composites [21,22,23,24,25]. The technique has also been successfully applied for the production of pectin films [26]. Compression molding, unlike the casting method, gives the possibility for the formation of 3D objects. This method has not been tested on apple pomace before.

The goal of the current study was to develop and evaluate bio-based films and 3D objects from apple pomace using solution casting and compression molding techniques, respectively. The new materials can potentially be used as disposable or edible packaging and tableware.

## 2. Materials and Methods

### 2.1. Materials

Apple pomace was kindly provided by Lyckans Äpple (Bredared, Sweden) which was stored at −20 °C until used. Other used materials in this study were glycerol (≥99.5%, Fisher BioReagents, Merelbeke, Belgium) and citric acid monohydrate (>99.5%, Duchefa Biochemie, Haarlem, The Netherlands).

### 2.2. Pretreatment of Apple Pomace

Apple pomace was either washed with water to remove free sugars and other soluble nutrients or not washed. The washing was carried out according to a previous report [5] with minor modifications. Cold water was used in the washing process to avoid starch dissolution. Initially, apple pomace was soaked overnight in cold tap water. The apple pomace to water ratio (kg/L) was 1:1.5 throughout the whole washing procedure. The water was then removed by manual pressing. The resulting material was washed two more times. In each washing step, the apple pomace was stirred in cold tap water for 10 min. A kitchen sieve was used to collect and rinse the remaining pomace. Both, the washed and the non-washed apple pomace were dried at 40 °C in a laboratory oven (Termaks, Bergen, Norway).

### 2.3. Formation of Apple Pomace Powder

A fine powder of the dried washed and non-washed apple pomace was obtained by a sequence of milling to sizes of 1.0 mm and 0.2 mm, using a variable speed rotor mill (Fritsch Pulverisette 14, Idar-Oberstein, Germany). To produce powders of even smaller particle sizes, approximately equivalent of using a 0.08 mm sieve, a ball mill was used (Retsch MM 400, Haan, Germany) in periods of 10 min at a frequency of 30 Hz.

### 2.4. Preparation of Films and 3D Objects from Apple Pomace

**Figure 1:**
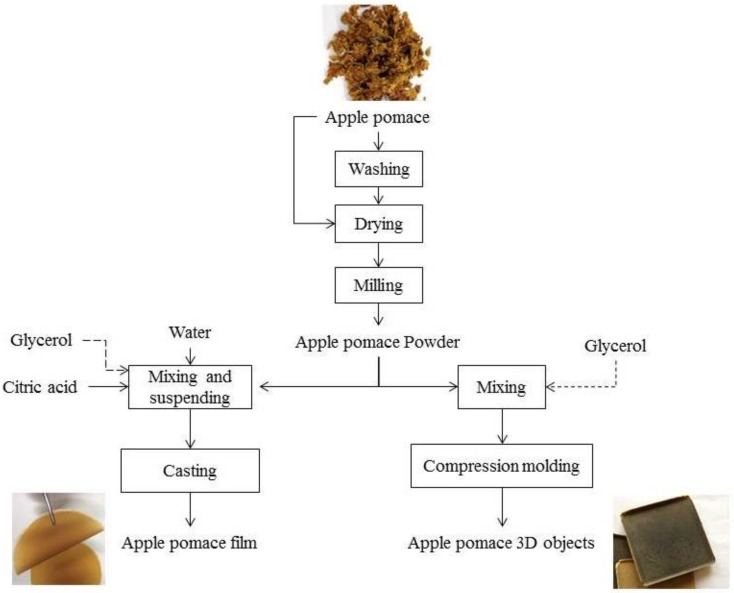
Flowchart of the methods used for production of bio-based films and 3D objects from apple pomace.

Solution casting and compression molding techniques were employed for production of bio-based films and 3D objects from apple pomace. The details of the methods are presented in Figure 1.

#### 2.4.1. Preparation of Bio-Based Films from Apple Pomace by Solution Casting Method

Formation of bio-based films was performed by modifying the method Bátori et al. [5] used for the formation of orange waste films. A mixture was prepared containing 2% (w/v) of washed apple pomace powder (particle size of approximately 0.08 mm) and 7% glycerol (w/w of apple pomace powder) dissolved in 1% (w/v) of citric acid solution. The mixture was made while heating to 70 °C under constant magnetic stirring at 560 rpm. By using a metal kitchen sieve, air bubbles were removed before 30 g of mixture was poured onto a non-sticky plate (Polytetrafluoroethylene, 100 mm in diameter) for casting. Bio-based films were made in triplicates. The plates were dried at 40 °C in a laboratory drying oven (Termaks, Bergen, Norway). The dry films were removed by gently pulling them off with pincers and stored in plastic zip bags until analyses. Additionally, two further mixtures of the non-washed apple pomace were prepared in the same way with or without the use of glycerol.

#### 2.4.2. Preparation of 3D Biomaterials from Apple Pomace by Compression Molding Method

Apple pomace powders of size 1.0 mm or 0.2 mm, either washed or not washed, were used for preparation of 3D objects. The apple pomace powders were either mixed with glycerol (apple pomace to glycerol ratio was 70:30) prior to compression molding according to Gurram et al. [26] or used directly. To fill the mold, 40 g of apple pomace–glycerol mixture or non-washed apple pomace without glycerol was placed into a 100 × 100 mm square mold. A 10-ton molding press (Rondol C2348, Stroke-on-Trent, UK) was used. A pressure of 8 MPa was applied for 20 min at 100 °C to form the 3D objects according to Gurram et al. [26]. The shaping mold was opened when it cooled to room temperature, and the fiberboard was removed and stored in plastic zip bags for further analyses.

### 2.5. Compositional Analyses of Apple Pomace

For compositional analyses, material recovery was performed as follows: The apple pomace was dried at 40 °C until constant weight. The moisture content of apple pomace was determined by a gravimetric method. Apple pomace was then soaked in distilled water overnight, taking the moisture content of the dry material into account, with apple pomace to water ratio of 1:1.5. Apple pomace was then collected via vacuum filtration using a grade three filter paper. This was then washed two more times, keeping the same ratio of apple pomace and water. The recovered solutions, after soaking and the two washing steps, containing soluble sugars and other solutes were analyzed for soluble sugars content as described in Section 2.5.3. Pectin extraction was performed directly after the last washing step (using the wet material) according to Section 2.5.1. The rest of the apple pomace was then dried at 40 °C and milled to a powder of 0.2 mm particle size. The apple pomace powder was then used for starch (Section 2.5.2), carbohydrate, and lignin analyses (Section 2.5.4).

#### 2.5.1. Pectin Content

High-performance microwave digestion system (Milestone Ethos UP MA182, Sorisole, Italy) was used for pectin extraction according to Bátori et al., with a minor modification, where the acidified water consisted of 7.5 mL of 0.1 M HNO_3_ and 37.5 mL of distilled water, in order for the mixture of 20 g apple pomace and 45 mL acidified water to reach pH 2. The rest of the procedure was performed in the same way [5]. An average of four measurements was reported as pectin content% (w/w).

#### 2.5.2. Starch Content

The total starch content of the apple pomace powder was determined according to Total Starch HK Assay Kit (K-TSHK, Megazyme, Bray, Ireland). The test was performed in duplicates and the average was reported.

#### 2.5.3. Sucrose, Fructose, and Glucose Content

The soluble sugars (i.e., sucrose, fructose, and glucose) of the apple pomace powder was determined according to Sucrose/D-Fructose/D-Glucose Assay Kit (K-SUFRG, Megazyme, Bray, Ireland), and high performance liquid chromatography (HPLC) (Waters 2695, Waters, Milford, USA) was used as control measurement for quantifying glucose and mixture of other sugars, using an analytical ion exchange column based on hydrogen ions (Aminex HPX-87H, Bio-Rad, Hercules, USA), operated at 60 °C with 0.6 mL/min of 5 mM H_2_SO_4_ as eluent. 

#### 2.5.4. Carbohydrate and Lignin Contents

Structural carbohydrates, containing arabinose, glucose, mannose, and xylose, as well as lignin contents of sugar-free apple pomace powder was determined according to NREL/TP-510-42618 [27], via two-step sulfur acid hydrolysis. The different fractions of carbohydrates were quantified with HPLC using a lead(II)-based column (HPX-87P, BioRad) with two Micro-Guard Deashing (Bio-Rad) pre-columns operated at 85 °C with 0.6 mL/min ultrapure water as eluent. The measurements were performed in triplicates and the averages were reported. Lignin content was determined following the same protocol in which acid soluble lignin was quantified by absorption (at 240 nm) and acid insoluble lignin was quantified by a gravimetric method.

### 2.6. Mechanical Testing of Bio-Based Films and Fiberboards

Dog-bone-shaped specimens were created by a laser cutting machine (GCC LaserPro Spirit GLS, New Taipei City, Taiwan) from the compression molded fiberboards and casted bio-based films. The specimens were analyzed for mechanical properties, according to ISO 527-1:1993 using a universal tester (Tinius Olsen H10KT, Horsham, PA, USA) and QMat software package. A moving cross-head was used to pull the specimens with a load cell of 100 N and a velocity of 10 mm/sec. An extensometer was used for the fiberboards to measure strain. The average of five specimens was reported as tensile strength (MPa) and elongation at maximum tensile strength (%).

### 2.7. Morphological Analyses of Bio-Based Films and Biocomposites

The morphology of the bio-based films and biocomposites was analyzed by a field emission scanning electron microscopy (FE-SEM) (Zeiss, Sigma, Jena, Germany). Surface and cross-sectional images of samples were taken after the samples were coated with gold. Photomicrographs were taken at 250 and 1.00 K x magnifications, using an accelerating voltage of 20.00 kV.

## 3. Results and Discussion

The need to replace conventional synthetic plastics has led to development of a rich and diverse field of research in bioplastic production. Production of bio-based materials from fruit wastes and fruit residues not only reduces the negative environmental effect of synthetic plastics but also contributes to the waste management issues. A wide variety of fruit waste has been tested for bioplastic production [7,28,29,30,31,32]. The entire fraction of apple pomace, without separation of seeds and stems or without the extraction of structural components, was used in the current study for the production of new bio-based films and 3D objects. The compositional analysis of the apple pomace is presented in Table 1.

Two typical approaches were used for preparation of films and 3D objects from apple pomace. The films were prepared getting benefit of the film forming ability of polysaccharides by solvent evaporation [33] in the casting process. As an alternative to the casting process, the self-binding ability of the biopolymers, using low pressure molding at high temperatures [24], was used for the development of apple pomace 3D objects without the use of any solvent or binder.

### 3.1. Production of Thin Films from Apple Pomace by Solution Casting

Both washed and non-washed apple pomace were used for film production by casting method according to Bátori et al. [5] (Figure 1). A brownish color with semi-transparent appearance was observed for apple pomace films regardless of preparation conditions. In general, the films were smooth and they did not contain holes as reported for orange waste films [5]. Presence of lignin in apple pomace, which is not present in orange waste, may be reasonable for a better quality of the apple pomace films regarding the absence of holes. The well-known adhesion properties of the lignin [34] is probably responsible for a better adhesion between the cellulose fibers and other carbohydrates in the apple pomace films. The thickness of the films was around 0.1 mm and was not affected significantly by the preparation conditions (Table 2).

#### 3.1.1. Morphology of Apple Pomace Films

**Figure 2:**
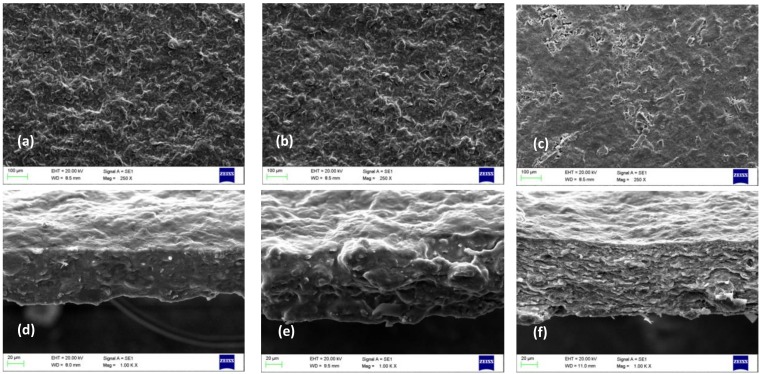
Bio-based films prepared from non-washed apple pomace, without (**a** and **d**) and with the use of glycerol (**b** and **e**), show a fluffy and more interconnected structure compared to bio-based films prepared from washed apple pomace with the use of glycerol (**c** and **f**). Images of (**a**), (**b**), and (**c**) are taken of the surfaces of films; while (**d**), (**e**), and (**f**) are cross-sectional images. Micrographs were taken respectively at 250 × and 1.00 K × magnifications using a 20.00 kV accelerating voltage.

In order to study the morphology of the films, FE-SEM images were taken from the surface and the cross-section of the films (Figure 2). Accordingly, the surface of the film made from washed apple pomace, using glycerol as plasticizer, was smoother than those prepared from non-washed apple pomace (Figure 2). This may indicate higher orientation degree of the fibers in the film. In contrast, the films made of non-washed apple pomace showed a fluffier structure, which was also confirmed by cross-section images. The film made of washed apple pomace had a compact layered structure (Figure 2f). In contrast, the films made of non-washed apple pomace exhibited a fluffy structure where the polysaccharide particles seemed to be more connected to each other, in the cross-sectional images (Figure 2d,e). This is probably because of the plasticizing effect of sugars as suggested by Vieira et al. [35] in starch- and protein-based thin films. The film prepared from the non-washed apple pomace, using glycerol as plasticizer, showed a swollen structure compared to the other films (Figure 2e). This is because of the presence of the glycerol, which acts as a secondary plasticizer in the system, making the movement of the particles much easier. Some studies have reported that in bio-based films, prepared from starch or gluten, the effect of the added plasticizer (glycerol) is strongly affected by naturally-occurring water (moisture content) [36,37]. This supported the assumption of a three-phase system of polysaccharides-sugar-glycerol that is collectively affecting the film properties. Moreover, the results indicate a stronger plasticizing effect of the sugars, compared to glycerol, in the two-phase systems of polysaccharides–sugars and polysaccharides–glycerol, as the films with glycerol and without sugar showed a non-connected, layered structure (Figure 2f).

#### 3.1.2. Mechanical Properties of the Apple Pomace Films

The mechanical properties of the films are presented in Table 2. The film prepared from washed apple pomace using glycerol as plasticizer exhibited the highest tensile strength at max and lowest elongation at max. This is also in agreement with the results of scanning electron microscopy where a layered morphology with higher fiber orientation was observed for this film (Figure 2f). The elongation of the non-washed films was significantly increased in the presence of sugars confirming the plasticizing effect of the sugars. The highest elongation (55%) was obtained for the films prepared from non-washed apple pomace and glycerol, indicating the effect of primary (sugar) and secondary (glycerol) plasticizers. The stronger plasticizing effect of the sugars, as predicted by FE-SEM images (Figure 2), was also confirmed here, as the film prepared from non-washed apple pomace was at least three times more flexible than the one prepared from washed apple pomace and glycerol (Table 1). 

Higher flexibility (elongation) was accompanied with a significant reduction in the mechanical strength of the films, which is reasonable and has also been also reported before by Cao et al. [38] for gelatin films and by Bourtoom et al. [39] for rice starch–chitosan films. 

### 3.2. Production of 3D Objects from Apple Pomace by Compression Molding

3D square shape objects (fiberboards) were developed from apple pomace using the compression molding technique according to Figure 1. Both washed and non-washed apple pomace were employed for production of fiberboards using this method. 

Thanks to the thermo-triggered self-binding ability of natural fibers, production of binder-less eco-composites from lignocellulosic materials has been reported in several studies [21,22,23,24,25]. Although the mechanism of the fiber binding at high temperature under pressure seems to be still unclear, in situ plasticization and lignin flow has been reported to be among the factors which facilitate the fiber binding at very high temperatures (200 °C) [21,23,25]. Although lignin is present in the apple pomace (Table 1), the plasticizing effect of lignin might not be very significant in the formation of apple pomace fiberboards, as the processing temperature was only 100 °C.

On the other hand, addition of plasticizers may lower the shaping temperature by increasing the mobility of the macromolecules and filling the voids [37], making the binding possible at lowered temperatures. Two plasticizers were tested in this study (i.e., glycerol and free sugars present in apple pomace). Glycerol has been previously used in several studies [26,37]. Dark brown sheets (10 x 10 cm) were formed from washed apple pomace (0.2 mm particle size) using glycerol as plasticizer, and the thickness of the sheet was 3.2 mm (Table 2). Darkening of gluten films was reported in the presence of glycerol when pressing temperatures were higher than 100 °C [37], and explained by the possible effects of higher network density, extensive aggregation, temperature effects of pigments, and Maillard reactions [40,41]. Apple pomace; however, does not contain gluten, but glycerol may have reacted with lignin under the applied conditions. To support this assumption, preliminary experiments resulted in a dark brown, spilled patch, instead of a firm sheet when lignin was mixed with glycerol applying the same preparation conditions. There is; however no evidence of lignin being responsible for the darkening of apple pomace fiberboards when glycerol was added, but it could be the case. 

Sugars have not been examined before for their plasticizing effect in binder-less lignocellulosic fiberboards. In this study the sugars available in the apple pomace were tested as plasticizer. Non-washed apple pomace (with particle size of 0.2 mm) was subjected to compression molding and resulted in light brown sheets with a thickness of 2.2 mm (Table 3). The difference between the thicknesses of the sheets made of washed and non-washed apple pomace indicate a more compact structure of the material made of non-washed apple pomace. This confirms the performance of sugars as plasticizer in formation of binder-less sheets from apple pomace. Moreover, the thickness of the sheet prepared from non-washed apple pomace with particle size of 1 mm was 2.9 mm, indicating a less compact structure due to the presence of bigger particles (Table 3).

#### 3.2.1. Morphology of Apple Pomace 3D Objects

**Figure 3:**
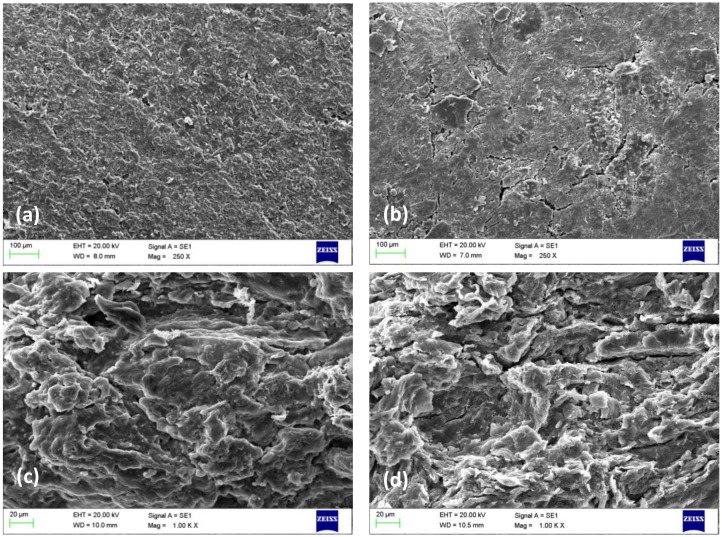
Fiberboards prepared from non-washed apple pomace without the addition of glycerol (**a** and **c**) show a more connected, smoother structure compared to the ones made from washed apple pomace with the addition of glycerol (**b** and **d**), which had cracks in the structure. Images of (**a**) and (**b**) are taken of the surfaces of specimens; while (**c**) and (**d**) are cross-sectional images. Micrographs were taken respectively, at 250 × and 1.00 K × magnifications using a 20.00 kV accelerating voltage.

Morphology of the 3D objects prepared from washed and non-washed apple pomace (0.2 mm particle size) was studied using scanning electron microscopy (Figure 3).

Similar to the films, the surface of the material prepared from washed apple pomace was smoother than the non-washed material (Figure 3a,b). This may indicate a higher orientation of the fibers in washed material and more flexibility of the fibers in non-washed material. However, more cracks were visible in the structure of the film made from the washed material (Figure 3b), which is indicating a better binding when the non-washed material was used with free sugars as plasticizer (Figure 3a). The cross-sectional images show somewhat a similar structure for both 3D objects, and some cracks were visible in the washed material (Figure 3d) compared to the unwashed (Figure 3c). Therefore, FE-SEM analysis confirms that slightly better binding was achieved in the presence of sugars as plasticizer compared to glycerol. 

#### 3.2.2. Mechanical Properties of the Apple Pomace 3D Objects

Fiberboards made of washed apple pomace and glycerol had a slightly higher tensile strength compared to the one made of non-washed, 5.8 and 3.7 MPa, respectively (Table 3). This is probably because of the higher orientation of the fibers in the washed biomaterial, which could be the result of the wetting effect of glycerol. The difference was; however, not as high as the one observed between different apple pomace films. This is because of the differences in the mechanism of the particle binding in the casting and compression molding methods. The tensile strength of the biomaterials prepared from non-washed apple pomace with 1 mm particle size was slightly higher than the one with 0.2 mm particle size, which indicates better binding of particles in the case of smaller particle sizes.

The modulus is a measurement of the stiffness and it follows the same pattern as the tensile strengths, with somewhat higher modulus for the washed biomaterial. However, specimens prepared of 0.2 mm particle size, without washing had slightly higher strength and lower modulus than the specimens of 1 mm particle size. Considering the standard deviations, the values for tensile strength and for modulus were not significantly different from each other.

Moreover, the elongations at max of the fiberboards were much lower compared to the films. This is due to the weaker binding of the particles in the compression molding compared to the solution casting method.

## 4. Conclusions

In this study, solution casting and compression molding techniques were successfully employed for preparation of biofilms and 3D objects from apple pomace, respectively. The choice of the plasticizer affected the characteristics of the products. The use of glycerol resulted in films and fiberboards with higher tensile strength. Using natural occurring sugars as plasticizer resulted in products with more connected structure; however, in the case of the films, it resulted in much higher elongation at maximum tensile strength. Finally, using apple pomace to produce bio-based films and 3D objects paves the way for producing environmentally-friendly materials that could both be a solution to the problem of plastic pollution and to apple pomace disposal. The new materials may be suitable for different applications including edible packaging and tableware.

## Figures and Tables

**Table 1:** Characterization of apple pomace. By washing of apple pomace, the free sugars and some other water-soluble components were removed, resulting in the sugar-free material.

Component		Proportion (%) ^1^
Recovery of sugar-free water insoluble fraction ^1^		39.41
Water insoluble fraction	Pectin ^2^	8.94 ± 1.20
	Starch ^2^	2.91 ± 0.00
	Cellulose ^2^	38.99 ± 0.42
	Hemicelluloses ^2^	29.42 ± 0.44
	Acid soluble lignin ^2^	6.51 ± 0.12
	Acid insoluble Lignin ^2^	16.43 ± 0.12
Water soluble fraction	Total free sugars ^1,3^	55.47 (50.39)
	Sucrose	17.53
	Fructose	26.92
	Glucose	11.01 (16.63)
Non-determined water-soluble fraction ^1,3^		5.12 (10.2)
Moisture in wet apple pomace		82.725 ± 0.07

^1^ Based on the dry apple pomace; ^2^ Based on the sugar-free dry material; ^3^ Values in parenthesis were determined by HPLC.

**Table 2:** Different preparation conditions and main properties of bio-based films prepared from apple pomace, using 1% (w/v) citric acid solution at 70 °C.

Washing Step	Glycerol (%)	Particle Size ^1^ (mm)	Thickness (mm)	Tensile Strength (MPa)	Elongation (%)
yes	7	~0.08	0.11 ± 0.01	16.49 ± 2.54	10.77 ± 3.19
no	7	~0.08	0.11 ± 0.01	3.27 ± 0.31	55.41 ± 5.38
no	0	~0.08	0.09 ± 0.00	4.20 ± 0.70	37.39 ± 10.38

^1^ Apple pomace particle size used for preparation of the films.

**Table 3:** Different preparation conditions and main features of fiberboards prepared from apple pomace applying 8 MPa pressure for 20 min at 100 °C.

Washing Step	Plasticizer (%)	Particle Size ^1^ (mm)	Thickness (mm)	Tensile Strength (MPa)	Elongation (%)	Young’s Modulus (MPa)
yes	Glycerol 30%	0.2	3.18 ± 0.07	5.79 ± 0.79	1.54 ± 0.09	633.4 ± 65.6
no	Free sugars ^2^	0.2	2.17 ± 0.51	3.71 ± 0.80	1.56 ± 0.13	367.1 ± 82.6
no	Free sugars ^2^	1	2.91 ± 0.02	3.02 ± 0.65	0.93 ± 0.21	485.7 ± 94.7

^1^ Apple pomace particle size used for preparation of fiberboards. ^2^ From non-washed apple pomace.
